# Cytosolic Superoxide Dismutase (SOD1) Is Critical for Tolerating the Oxidative Stress of Zinc Deficiency in Yeast

**DOI:** 10.1371/journal.pone.0007061

**Published:** 2009-09-16

**Authors:** Chang-Yi Wu, Janet Steffen, David J. Eide

**Affiliations:** Department of Nutritional Sciences, University of Wisconsin-Madison, Madison, Wisconsin, United States of America; Newcastle University, United Kingdom

## Abstract

Zinc deficiency causes oxidative stress in many organisms including the yeast *Saccharomyces cerevisiae*. Previous studies of this yeast indicated that the Tsa1 peroxiredoxin is required for optimal growth in low zinc because of its role in degrading H_2_O_2_. In this report, we assessed the importance of other antioxidant genes to zinc-limited growth. Our results indicated that the cytosolic superoxide dismutase Sod1 is also critical for growth under zinc-limiting conditions. We also found that Ccs1, the copper-delivering chaperone required for Sod1 activity is essential for optimal zinc-limited growth. To our knowledge, this is the first demonstration of the important roles these proteins play under this condition. It has been proposed previously that a loss of Sod1 activity due to inefficient metallation is one source of reactive oxygen species (ROS) under zinc-limiting conditions. Consistent with this hypothesis, we found that both the level and activity of Sod1 is diminished in zinc-deficient cells. However, under conditions in which Sod1 was overexpressed in zinc-limited cells and activity was restored, we observed no decrease in ROS levels. Thus, these data indicate that while Sod1 activity is critical for low zinc growth, diminished Sod1 activity is not a major source of the elevated ROS observed under these conditions.

## Introduction

Zinc is an essential nutrient because it is a required structural or catalytic cofactor for many proteins. These proteins include zinc finger-containing transcription factors and enzymes such as carbonic anhydrase, alcohol dehydrogenase, and Cu/Zn-superoxide dismutase. Excess zinc can also be toxic to cells. Zinc toxicity may be due to the binding of zinc to inappropriate sites that inhibit enzyme function or by displacing other metal ions from the active sites of enzymes. Thus, mechanisms of zinc homeostasis are required to maintain intracellular zinc levels within a narrow optimal range.

Both extremes of zinc status, i.e. excess and deficiency, cause oxidative stress in cells through the increased accumulation of reactive oxygen species (ROS) such as superoxide anion, hydrogen peroxide, and hydroxyl radical [1], [2]. Zinc excess likely leads to increased levels of ROS by disrupting mitochondrial function and inhibiting the activity of the electron transport chain [3], [4]. The mechanisms underlying the increased oxidative stress that occurs in zinc deficiency are much less clear. Possible mechanisms include loss of activity of zinc-dependent antioxidant enzymes such as superoxide dismutase and loss of the antioxidant properties of zinc-metallothionein complexes [5], [6]. Whatever the source of oxidative stress, zinc deficiency causes increased levels of lipid and protein oxidation [7], [8]. In addition, the oxidative stress of zinc deficiency leads to increased levels of DNA damage [8], [9], [10]. For these reasons, zinc deficiency has been proposed to be an important risk factor for cancer and other human diseases [5], [11]. It has been estimated that ∼2 billion people worldwide do not consume adequate levels of zinc in their diets so zinc deficiency is clearly an important public health problem [12].

To combat oxidative stress, organisms express a large repertoire of different antioxidant proteins. These include thioredoxin-dependent peroxiredoxins, superoxide dismutases, glutathione peroxidases, and catalases. These proteins play different roles in metabolizing ROS depending on the specific form of ROS on which they act, the compartment in which they are targeted, and how their expression or activity is regulated in response to oxidative stress and other environmental signals. We recently showed that yeast, like other organisms, has elevated ROS when grown under zinc-limiting conditions [13]. We also found that these cells increase expression of the major cytosolic peroxiredoxin, Tsa1, to eliminate at least some of that increased ROS. Tsa1 degrades H_2_O_2_ and this gene is critical for the growth of zinc-limited yeast. Moreover, *TSA1* gene expression is induced in zinc-limited cells by the action of the Zap1 transcription factor [13], [14]. Zap1 up-regulates expression of plasma membrane and organellar zinc transporters to maintain zinc homeostasis and induces expression of other genes, such as *TSA1*, to help cells adapt to the stresses that accompany zinc deficiency [15].

In this report, we describe a search for other antioxidant systems that are important for growth under zinc-limiting conditions. As a result, we have discovered that the activity of cytosolic Cu/Zn-superoxide dismutase, encoded by the *SOD1* gene, is also critical for zinc-limited growth. Sod1's copper chaperone Ccs1 is also required for zinc-limited growth consistent with its role in activating Sod1. To our knowledge, this is the first demonstration of the critical role of Cu/Zn-SOD and its copper chaperone in zinc deficiency. Coupled with previous studies showing Sod1 is required for tolerance of high zinc [16], our results show that Sod1 is required for growth at both extremes of zinc status.

## Materials and Methods

### Yeast strains, plasmid and growth conditions

Yeast cells were grown in YPD (YP medium + 2% glucose) and in synthetic defined SD medium with 2% glucose and any necessary auxotrophic requirements. Methionine, leucine, and lysine were supplemented into all cultures except when used as a plasmid retention marker. YPD and SD are zinc-replete because they contain micromolar levels of zinc and lack strong zinc chelators. Yeasts were made zinc limited by culturing in low zinc medium (LZM) prepared as described previously [17]. LZM is zinc limiting because it contains 1 mM EDTA and 20 mM citrate to buffer metal availability. Zinc was added as ZnCl_2_. Cells were grown anaerobically using the BBL GasPak™ system (Pharmingen). Strains used in this study were DY1457 (*MATα ade6 can1 his3 leu2 trp1 ura3*), CWY2 (*MATα his3 leu2 ura3*), CWY8 (CWY2 *tsa1Δ::kanMX4* ), JSY92 (CWY2 *sod1Δ::hphMX4*), JSY94 (CWY2 *tsa1Δ::kanMX4 sod1Δ:: hphMX4*), BY4743 (*MATa/MATα his3/his3 leu2/leu2 ura3/ura3 lys2/+ met15/+*), BY4743 *sod1Δ::kanMX4/sod1Δ::kanMX4* and BY4743 *ccs1Δ::kanMX4/ccs1Δ::kanMX4*. Strains used in the initial screen were isogenic to BY4743 and carried the indicated homozygous mutation tagged with the *kanMX4* cassette (Invitrogen). Plasmids used in this study were as follows: pEL124 (cytSOD2) encodes a cytosolic-targeted form of *S. cerevisiae* Mn-dependent Sod2 and was a gift from Dr. Val Culotta (Johns Hopkins University) [18]. YEp351 is a high copy vector with a *LEU2* selectable marker. YEp600 contains the wild-type *SOD1* gene in YEp351 expressed from its own promoter. Plasmid YEp351-yH46C encodes a mutant allele in which histidine 46 was replaced with cysteine in YEp600 [16]. YEp600 and YEp351-yH46C were gifts from Dr. Joan Valentine (University of California-Los Angeles).

### RNA and protein analysis

S1 nuclease protection assays were performed with total RNA as described [13]. For each reaction, 15 µg of total RNA was hybridized to a^ 32^P-end-labeled DNA oligonucleotide probe before digestion with S1 nuclease and separation on a 10% polyacrylamide gel containing 5M urea. Band intensities were quantitated by PhosphorImager analysis (PerkinElmer Life Sciences).

### SOD activity assays and immunoblotting

Cell lysates for SOD activity assays were prepared as described [18], [19]. Briefly, cells were harvested at OD_600_ of 1.0 and were lysed using glass beads in 0.5 ml lysis buffer [10 mM NaPO_4_ (pH 7.8), 5 mM EDTA, 0.1% Triton, 50 mM NaCl, 0.5 mM phenylmethylsulfonyl fluoride (PMSF) and Complete EDTA-free Protease Inhibitor cocktail (Roche Applied Science)]. The cell suspensions were vortexed 5×1 min with 1 min intervals on ice. Cell debris and glass beads were removed by centrifugation at 13,000×*g* for 10 min at 4°C. The supernatant was collected and protein concentration was measured by the Bradford method [20]. To perform the SOD activity assays, extracts were separated on a nondenaturing 12% polyacrylamide gel. After electrophoresis, the gel was subjected to nitro blue tetrazolium (NBT) staining for detecting superoxide. A 75-ml staining solution contained 10 mg NBT (Sigma) in 50 mM KPO_4_ (pH 7.8), 0.1 mg/ml riboflavin, and 1 µl/ml TEMED. The gels were soaked in the dark in staining solution for 45 min and then exposed to light for color development. For immunoblotting, cell lysates were separated on denaturing 12% SDS-polyacrylamide gels prior to blotting. The primary antibodies used were anti-Sod1 (a gift from Dr. Valeria Culotta, Johns Hopkins University) and anti-Pgk1 (Molecular Probes). Signal intensities of activity and protein were quantified using OPTI-QUANT software.

### DCF Assays

Measurement of the production of the reactive oxygen species (ROS) used 2, 7-dichlorodihydrofluoroscein diacetate (DCFH-DA) (Calbiochem) [13]. DCFH-DA is membrane-permeable and is trapped intracellularly following deacetylation. The resulting compound, DCFH, reacts with ROS to produce the oxidized fluorescent form, 2,7-dichlorofluoroscein (DCF). ROS analysis using DCFH-DA was performed as follows. Yeast cells grown to an OD_600_ of 0.6–0.8 were treated in the dark with 10 µM DCFH-DA in culture media for 1 h prior to harvesting. Cells were then washed twice in ice-cold phosphate-buffered saline, resuspended in phosphate-buffered saline, and disrupted by vortexing with glass beads. Following centrifugation at 13,000×*g* for 10 min at 4°C, the supernatant was collected, and protein concentration was measured by the Bradford method. DCF fluorescence intensity was measured at an excitation wavelength of 504 nm and an emission wavelength of 524 nm and then normalized to protein level.

## Results

### Requirement for Cu/Zn-superoxide dismutase (SOD1) in low zinc

**Figure 1:**
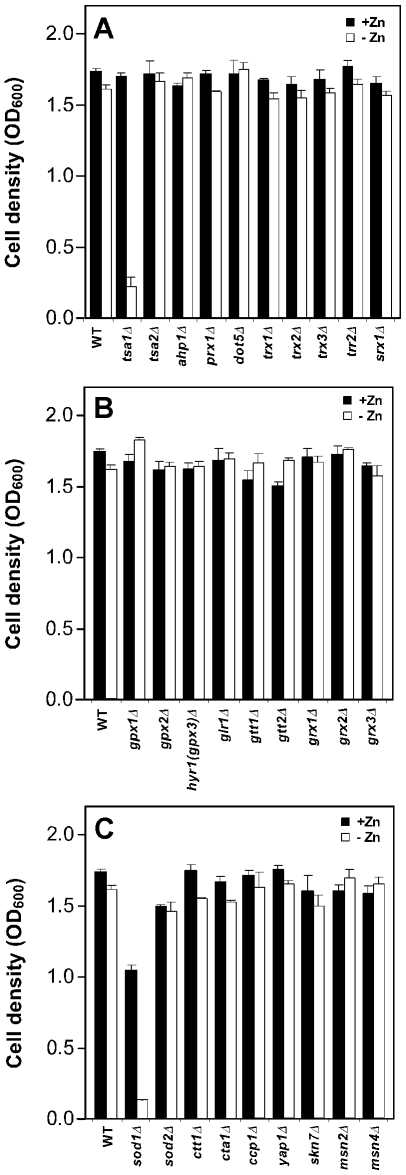
The importance of antioxidant genes to growth under zinc-limiting conditions. Wild type (WT, BY4743) cells and isogenic mutants defective for various antioxidant proteins were inoculated into LZM medium supplemented with 1000 µM (+Zn) or 1 µM (–Zn) ZnCl_2_ at an initial optical density measured at 600 nm (OD_600_) of 0.01 and 0.02, respectively. The cultures were then incubated at 30°C with aeration and their optical densities were determined after 40 h. A) Mutants defective in peroxiredoxins or other thioredoxin-related antioxidant proteins. B) Mutants defective in glutathione peroxidases or other glutathione-related antioxidant proteins. C) Mutants defective in superoxide dismutases, catalases, cytochrome c peroxidase, or oxidative stress-responsive transcription factors. The plotted values represent the means of three independent cultures and the *error bars* indicate 1 S.D.

We previously observed that zinc-deficient yeast experience increased oxidative stress and that the Tsa1 peroxiredoxin is required to deal with this oxidative stress and allow for optimal growth under zinc-limiting conditions [13]. These results raised the question of whether Tsa1 was uniquely important in this role or whether other antioxidant genes were also important for low zinc growth. To address this question, we assessed the growth of strains mutated for 28 different antioxidant genes for growth in low and high zinc. Wild type cells and deletion mutants were inoculated into high (LZM + 1000 µM ZnCl_2_) and low zinc (LZM + 1 µM ZnCl_2_) liquid medium at low initial cell density and cultured for 48 h prior to measuring the cell densities of the cultures. The tested genes involved in thioredoxin-dependent antioxidant pathways encode five peroxiredoxins (*TSA1, TSA2, AHP1, PRX1, DOT5*), thioredoxins (*TRX1-3*), thioredoxin reductase (*TRR2*) and sulfiredoxin (*SRX1*). Of these, only *TSA1* was required for low zinc growth (**Fig. 1A**). A second major group of antioxidants are the glutathione-dependent systems. The genes tested from this group encoded glutathione peroxidases (*GPX1-3*), glutathione reductase (*GLR1*), glutathione S-transferases (*GTT1-2*), and glutaredoxins (*GRX1-3*). None of these genes were required for low zinc growth (**Fig. 1B**). Additional antioxidant systems include superoxide dismutases (*SOD1*, *SOD2*), catalases (*CTT1*, *CTA1*), and cytochrome c peroxidase (*CCP1*). Of these, only the *sod1Δ* mutant showed a strong growth defect in low zinc (**Fig. 1C**). Unlike the *tsa1Δ* mutant, which grew as well as wild type cells in high zinc, the *sod1Δ* mutant showed reduced growth (∼60% of wild type). Slower growth of the *sod1Δ* mutant in zinc-replete aerobic conditions has been observed previously [21]. However, in low zinc, both *tsa1Δ* and *sod1Δ* strains showed only about 10% of the growth yield observed in zinc-replete cultures. Finally, none of the transcription factors involved in oxidative stress response that we tested (*YAP1, SKN7, MSN2, MSN4*) were defective for low zinc growth. Thus, among the many antioxidant genes examined, *SOD1* and *TSA1* play the key roles in supporting zinc-limited growth.

### The growth defect of sod1Δ mutants in low zinc medium is zinc-specific

**Figure 2:**
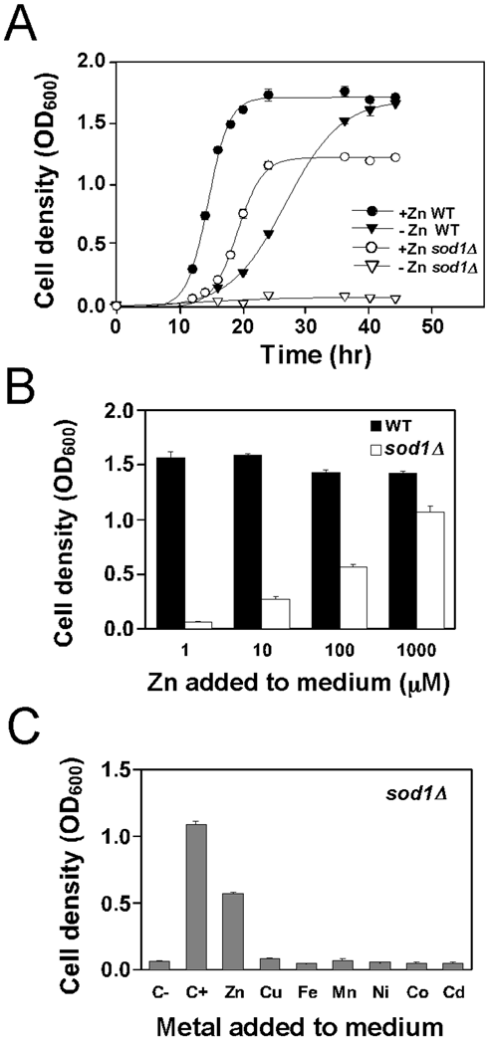
*SOD1* is required for growth in zinc-limiting conditions. A) Wild type (WT, BY4743) and *sod1Δ* mutant (BY4743 *sod1Δ*) cells were inoculated into LZM medium supplemented with 1000 µM (+Zn) or 1 µM (–Zn) ZnCl_2_ at an initial OD_600_ of 0.02. The cultures were then incubated at 30°C with aeration and their optical densities were measured over time. B) The same strains as in panel A were inoculated into LZM supplemented with the indicated concentration of ZnCl_2_ at a starting OD_600_ of 0.02. The cultures were incubated at 30°C with aeration for 48 h prior to measuring their optical densities. C) *sod1Δ* mutant cells were inoculated into LZM + 1 µM ZnCl_2_ medium with or without 100 µM of the indicated metal ion added as the chloride salt. The negative control condition (C-) had no metals added and the positive control (C+) had 1000 µM ZnCl_2_ added. Cultures were inoculated at an initial OD_600_ of 0.01 and their optical densities were determined after 48 h. The values plotted in panels A, B and C are the means of three independent cultures and the *error bars* indicate 1 S.D.

To further characterize the effect of *sod1Δ* mutation on zinc-limited growth, wild type and *sod1Δ* mutant cells were inoculated into zinc-replete or zinc-limiting media and cell growth was monitored over time. Both wild type and *sod1Δ* mutant cells grew well when inoculated into zinc-replete medium (LZM + 1000 µM ZnCl_2_) (**Fig. 2A**). Zinc-replete *sod1Δ* mutants showed a longer lag phase and reached saturation at a lower cell density than the wild type cells. While growth of wild type cells was somewhat impaired in the zinc-limiting medium (LZM + 1 µM ZnCl_2_) due to zinc deficiency, the *sod1Δ* mutant was severely defective for growth under this condition. It should be noted that *sod1Δ* mutants are auxotrophic for methionine, leucine, and lysine due to the ROS-sensitive biosynthetic pathways for these amino acids [21]; the media in these experiments were supplemented with these amino acids so the poor growth observed is not due to these auxotrophies.

To determine the level of zinc required for optimal growth of the *sod1Δ* mutant, wild type and *sod1Δ* cells were inoculated into LZM supplemented over a range of zinc concentrations and cell density was determined after 48 h. While the poor growth of the *sod1Δ* mutant was again observed in LZM + 1 µM ZnCl_2_, supplementing with higher concentrations improved growth of this mutant strain considerably (**Fig. 2B**). However, even zinc supplements as high as 100 µM did not restore growth to their maximum zinc-replete level. These results indicate that *sod1Δ* mutants are sensitive to even mild deficiency. As shown in **Fig. 2C**, the growth defect was specific to zinc deficiency; supplementation with 100 µM concentrations of several other metal ions did not restore growth to the *sod1Δ* mutant strain.

### Growth in zinc-limiting conditions requires Sod1 activity

**Figure 3:**
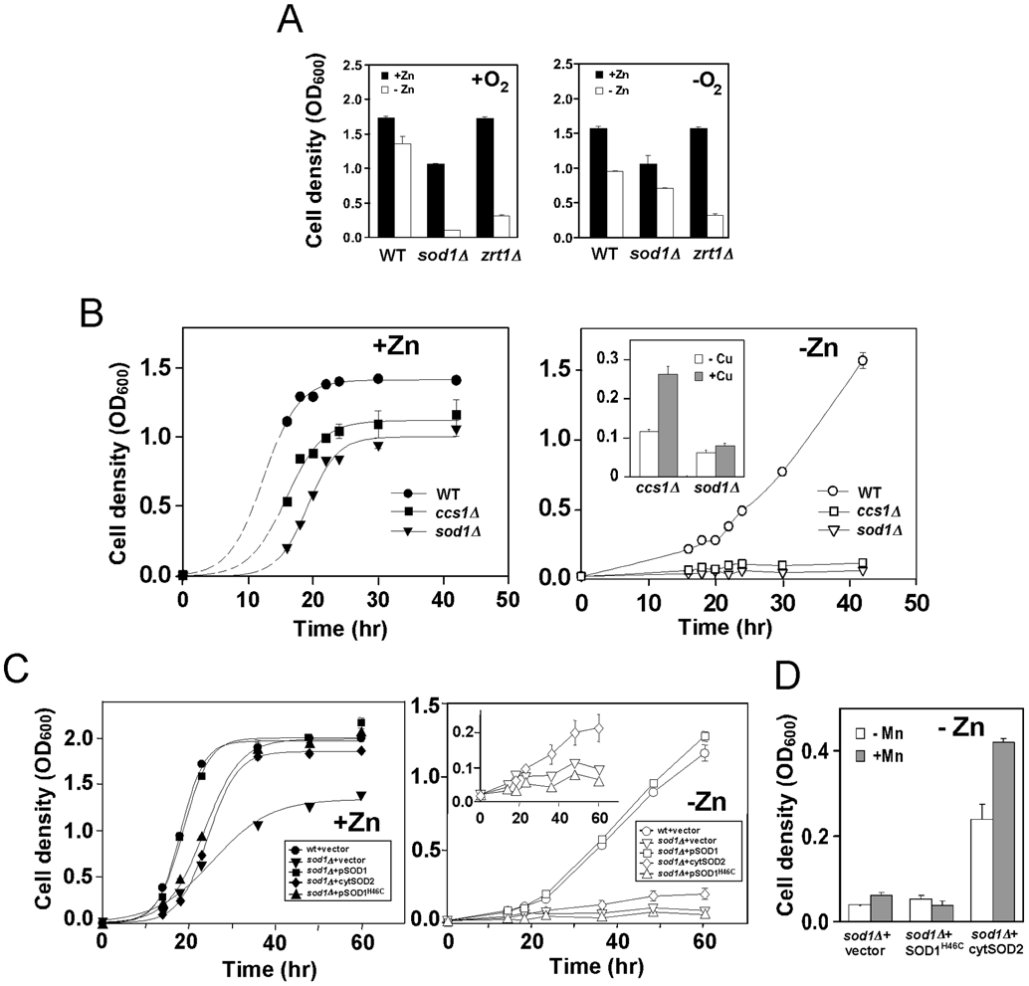
Sod1 activity is required to deal with the oxidative stress of zinc deficiency. A) Anaerobic conditions suppress the *sod1Δ* growth defect in low zinc. Cells of the indicated genotype were inoculated at an initial OD_600_ of 0.01 in high zinc (+Zn, LZM + 1000 µM ZnCl_2_) or 0.04 in low zinc medium (–Zn, LZM + 1 µM ZnCl_2_) under aerobic (+O_2_) or anaerobic (–O_2_) conditions. Culture optical densities were then measured after 20 h of incubation at 30°C. B) *CCS1* is required for low zinc growth. Wild type (WT, BY4743), *sod1Δ* (BY4743 *sod1Δ*) and *ccs1Δ* (BY4743 *ccs1Δ*) mutant cells were inoculated into high zinc (+Zn, LZM + 1000 µM ZnCl_2_, *left panel*) or low zinc (-Zn, LZM + 1 µM ZnCl_2_, *right panel*) at an initial OD_600_ of 0.02. The cultures were incubated at 30°C with aeration and their optical densities were monitored over time. The curve fitting for the +Zn cultures was performed with SigmaPlot software and the *dashed* lines indicate the early predicted values. As shown in the *inset*, *sod1Δ* and *ccs1Δ* strains were inoculated into low zinc media (LZM + 1 µM ZnCl_2_) with or without 100 µM added copper (CuCl_2_). Culture optical densities were measured after 42 h of incubation at 30°C. C) Wild type (BY4743) or *sod1Δ* (BY4743 *sod1Δ*) mutant cells bearing the indicated plasmids were inoculated into high (+Zn, LZM + 1000 µM ZnCl_2_, *left panel*) or low zinc (-Zn, LZM + 1 µM ZnCl_2_, *right panel*) and optical densities were measured over time. D) *sod1Δ* mutant cells bearing the indicated plasmid were cultured in low zinc media (LZM + 1 µM ZnCl_2_) with or without 100 µM added manganese (MnCl_2_). Culture optical densities were measured after 60 h of incubation at 30°C. The data in all panels represent the means three independent cultures and the *error bars* indicate 1 S.D.

To investigate the functional role of Sod1 in zinc-limited cells, we examined whether Sod1 activity was required to combat the oxidative stress of zinc deficiency. We predicted that growth under anaerobic conditions, under which the generation of reactive oxygen species is diminished, would suppress the *sod1Δ* mutant growth defect. As shown in **Fig. 3A**, this was indeed the case. While growth of wild type cells was not greatly altered by anaerobic growth, anaerobic *sod1Δ* mutants grew almost as well in low zinc as they did in the zinc-replete medium. An alternative explanation for these results is that cells grown under anaerobic conditions may have a lower requirement for zinc than do aerobically grown cells. If this were the case, the improved growth of *sod1Δ* mutants under anaerobic conditions would be due to zinc repletion rather than decreased oxidative stress. However, a *zrt1Δ* mutant defective for zinc uptake grew poorly under low zinc conditions under both aerobic and anaerobic conditions (**Fig. 3A**) suggesting that zinc requirements were not greatly altered by anaerobiosis. In addition, wild type cells showed a similar dose response of growth to added zinc under aerobic and anaerobic conditions indicating that zinc requirement of yeast cells is not greatly altered by the absence of oxygen (data not shown).

A second prediction was that zinc-limited growth would also require the Ccs1 copper chaperone. Ccs1 is needed to deliver copper to yeast Sod1 for its activity [22]. While *ccs1Δ* mutants grew relatively well in zinc-replete cells, these mutants were severely defective for zinc-limited growth (**Fig. 3B**). The low zinc growth defect of *ccs1Δ* mutants could be partially suppressed by supplementation with copper (**Fig. 3B**, *inset*); it has been shown previously that high copper can weakly bypass the requirement of Sod1 for Ccs1 copper delivery [23]. However, copper supplements had no effect on *sod1Δ* low zinc growth.

To further investigate whether activity of Sod1 is required for low zinc growth, we tested whether a zinc-binding but catalytically inactive allele, Sod1^H46C^, or mitochondrial manganese-dependent Sod2 could suppress the low zinc growth defect when targeted to the cytosol. The Sod1^H46C^ allele was of particular interest because it was previously shown to confer tolerance of *sod1Δ* cells to high zinc [16]. In zinc-replete cells, both Sod1^H46C^ and cytosolic Sod2 partially suppressed the growth defect of *sod1Δ* mutant cells (**Fig. 3C**). This result suggests that the Sod1^H46C^ allele retains some residual SOD activity. In low zinc, however, expression of Sod1^H46C^ had no beneficial effects on cell growth (**Fig. 3C**). Expression of the manganese-dependent Sod2 in the cytosol resulted in weak but detectable suppression of the low zinc growth defect of the *sod1Δ* mutant (**Fig. 3C**, *inset*). Additional manganese improved the ability of cytosolic Sod2 to suppress the *sod1Δ* mutation (**Fig. 3D**) suggesting that the weak suppression is due to inefficient metallation of the Sod2 protein in the cytosol. Manganese supplements alone are known to suppress the phenotypes of *sod1Δ* mutants [24] but no benefit was seen with vector-transformed or Sod1^H46C^-expressing cells. Taken together, the results shown in **Figure 3** indicate that Sod1 activity is required for zinc-limited growth. This is in contrast to the observation that catalytically inactive Sod1 suppresses zinc toxicity.

### Sod1 activity is diminished in zinc-limited cells

**Figure 4:**
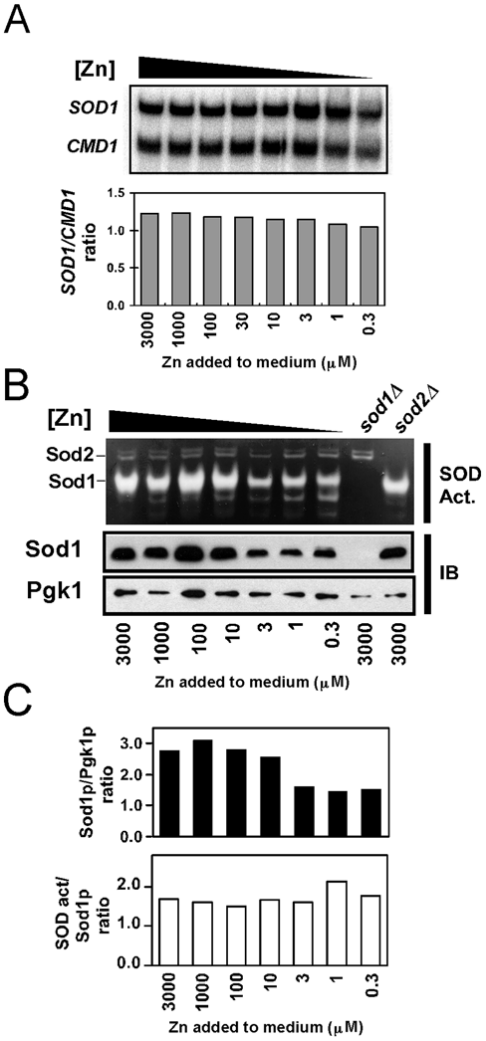
Effects of low zinc growth on Sod1 expression and activity. A) Wild type cells were grown in LZM supplemented with the indicated zinc concentrations. RNA was then isolated and analyzed for *SOD1* and *CMD1* mRNA levels by S1 nuclease protection assay. The ratio of *SOD1* mRNA to *CMD1* mRNA was determined in two independent experiments with similar results. B) Sod1 protein accumulation and activity were assayed in wild type, *sod1Δ*, and *sod2Δ* mutant cells grown in LZM supplemented with the indicated zinc concentrations. Sod1 activity was assessed using an in-gel assay (SOD Act.) and Sod1 protein accumulation was assessed by immunoblotting (IB). Pgk1 phosphoglycerate kinase was used as an immunoblot loading control. The results shown in panel B are representative of two independent experiments. C) The Sod1 protein levels were normalized to loading control Pgk1 protein and plotted in the histogram. Relative specific activities of Sod1 were assessed by dividing the measured Sod1 activities by the corresponding Sod1 protein levels.

Sod1 is a zinc-dependent enzyme. Because of this fact, one of the proposed mechanisms for the source of oxidative stress under zinc deficiency is loss of Sod1 activity and the consequent build up of ROS that is constitutively generated by aerobic metabolism [5]. To investigate this hypothesis, we first examined the effects of zinc deficiency on *SOD1* expression, protein accumulation, and SOD activity. As shown in **Fig. 4A**, zinc deficiency had little impact on *SOD1* mRNA levels. This was especially clear when *SOD1* mRNA levels were normalized to calmodulin (*CMD1*) mRNA that was used as a loading control. In contrast, Sod1 protein accumulation was reduced by ∼50% under zinc-limiting conditions (**Fig. 4B**). Sod1 activity decreased to a similar degree under zinc deficiency. No effect of zinc limitation was apparent on the activity of manganese-dependent Sod2 activity. Quantitation of Sod1 protein and activity levels suggested that the protein that does accumulate in zinc-limited cells is fully active, albeit less abundant than in zinc-replete cells (**Fig. 4C**).

**Figure 5:**
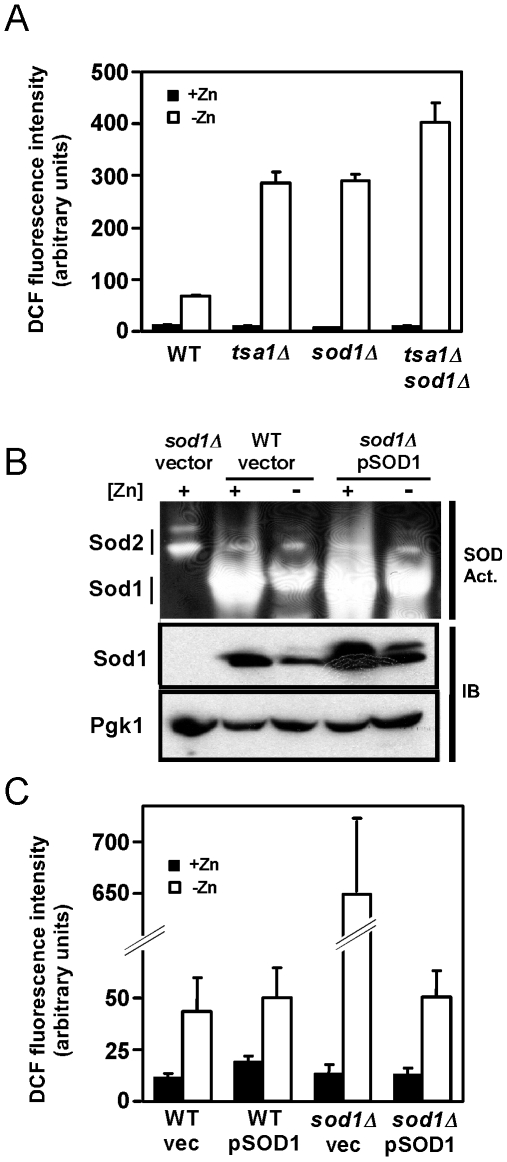
The effect of *SOD1* mutation and overexpression on oxidative stress. A) Wild type (WT, CWY2), *tsa1Δ* (CWY8), *sod1Δ* (JSY92), and *tsa1Δ sod1Δ* (JSY94) were inoculated into high zinc (+Zn, LZM + 1000 µM ZnCl_2_) or low zinc (-Zn, LZM + 1 µM ZnCl_2_) at an initial OD_600_ of 0.02. The cultures were incubated at 30°C with aeration for 24 h and then harvested and then assayed for ROS levels using DCFH-DA. B) Wild type or *sod1Δ* mutants were transformed with either the YEp351 vector or YEp600 (pSOD1), a high copy plasmid that results in overexpression of Sod1. The cells were grown as described for panel A and then assayed for Sod1 activity (SOD Act.) and protein level (IB) as described in Figure 4. The results indicate that YEp600 increases Sod1 activity in low zinc. C) Wild type and *sod1Δ* mutants transformed with either YEp351 (vec) or YEp600 (pSOD1) were grown as described for panel A and assayed for ROS levels using DCFH-DA. The data in panels A and C represent the means three independent cultures and the *error bars* indicate 1 S.D.

The decreased Sod1 in zinc-limited cells is consistent with the hypothesis that the loss of SOD activity leads directly to the increased oxidative stress detected in these cells. This hypothesis is also consistent with our previous observation that increased ROS in wild type cells occurs under the same conditions where we observed loss of Sod1 activity (LZM + 3 or less µM Zn) [13]. If so, we predicted that ROS levels would be elevated in zinc-limited *sod1Δ* mutants. ROS was detected in these experiments using DCFH-DA. DCFH fluorescence increases when it is oxidized to DCF by O_2_^−^, H_2_O_2_, or ^.^OH [25]. As shown in **Fig. 5A**, all strains tested had little DCFH-detectable ROS in zinc-replete conditions. As we observed previously, ROS levels rose ∼10 fold in zinc-limited wild type cells and ∼40-fold in the zinc-limited *tsal1Δ* mutant. Thus, loss of Tsa1 activity greatly increases oxidative stress in zinc-limited cells. Similarly, ROS levels in the zinc-limited *sod1Δ* mutant also rose to very high levels. When both mutations were combined, even higher levels of ROS were detected. These results indicate that, like Tsa1, Sod1 acts on ROS in zinc-limited cells.

To determine whether decreased Sod1 activity in zinc-limited wild type cells is indeed the source of the increased ROS in these cells, we overexpressed Sod1 from a high copy plasmid. We predicted that if loss of Sod1 activity was the source of the ROS, restoration of that activity by overexpression would result in ROS levels similar to zinc-replete cells. Control experiments indicated that overexpression of Sod1 greatly elevated its activity in zinc-limited cells indicating that the higher expressed level of Sod1 protein could compete effectively for the small amount of available zinc in these cells (**Fig. 5B**). When Sod1-overexpressing cells and vector-transformed control cells were assayed for ROS levels, however, we found that cells with increased Sod1 activity did not have reduced levels of ROS (**Fig. 5C**). These results suggest that the loss of Sod1 activity in zinc-limited cell is not responsible for the increased oxidative stress that accumulates under these conditions.

## Discussion

We describe here a screen for antioxidant genes in addition to *TSA1* that are important in combating the oxidative stress that occurs in zinc-limited cells. This analysis identified *SOD1*, encoding cytosolic Cu/Zn-superoxide dismutase, as critically important for low zinc growth. Sod1 was shown previously to be important for tolerating such varied stresses as oxidant insult, heat shock, hyperosmosis, freeze-thaw stress, stationary phase growth arrest, and ER stress [26], [27], [28], [29], [30], [31]. To our knowledge, this is the first demonstration of the essential role that Sod1 plays in supporting zinc-limited growth.

It was previously found that Sod1 is also required for resistance to high zinc [16]. Thus, Sod1 is required at both extremes of zinc status. Intriguingly, despite the fact that high zinc induces oxidative stress, the contribution of Sod1 to zinc tolerance is not dependent on its SOD activity and therefore does not involve Sod1's antioxidant role. A zinc binding but catalytically inactive Sod1 allele, Sod1^H46C^, conferred wild type zinc tolerance on a *sod1Δ* mutant strain while an active, Mn-binding SOD did not [16]. These observations led to the hypothesis that Sod1 serves as a “zinc sink” and, by binding zinc in the cell, helps buffer cytosolic zinc and thereby contributes to zinc tolerance. It has been estimated that there are ∼500,000 molecules of Sod1 protein per cell under zinc-replete conditions [32] so the amount of zinc that is bound by this one protein is substantial. These observations initially suggested to us that Sod1 may be playing a similar role in low zinc by serving as a zinc reservoir and supplying zinc to other proteins under those conditions. However, our results indicate that Sod1 enzymatic activity is required in low zinc suggesting that it is needed to metabolize increased levels of superoxide anion that accumulate in these cells. The source of ROS in low zinc is still unknown and this is an issue we are currently addressing. Our results indicate that decreased Sod1 activity is not the primary source. Other possible sources under investigation include mitochondrial dysfunction and/or ER stress.

Sod1 and the Tsa1 peroxiredoxin are both critical for low zinc growth. Sod1 converts O_2_^−^ into H_2_O_2_ (and O_2_) while Tsal metabolizes H_2_O_2_ into H_2_O. Thus, it is conceivable that these two proteins function primarily in a sequential manner with Sod1 generating H_2_O_2_ and Tsa1 further reducing that oxidant. If true, this would suggest that O_2_^−^ is the primary form of ROS generated in zinc-limited cells. This hypothesis remains to be tested. However, our observation that the *tsa1Δ sod1Δ* double mutant has elevated ROS when compared to either single mutant suggests that these proteins are not functioning solely in a sequential fashion. It is intriguing that substantially higher amounts of zinc in the growth medium are required to suppress the *sod1Δ* mutant than the *tsa1Δ* mutant; the low zinc growth defect of the *tsa1Δ* mutant was suppressed by as little as 10 µM added zinc [13] while the *sod1Δ* mutant required greater than 10-fold more zinc for suppression. While the mechanism underlying this difference is unknown, it clearly shows that Sod1 is needed for optimal cell growth during even modest zinc limitation while Tsa1 is only required under the most severely zinc-deficient conditions.

We noted that Sod1 activity is decreased by about 50% in zinc-limited cells. Protein levels also decreased to a similar extent despite no change in mRNA levels. These results suggest that if Sod1 fails to acquire its zinc cofactor soon after translation, the protein is degraded. While the mechanism of Sod1 turnover in yeast has not been studied, both proteasomal and autophagic degradation of SOD has been observed in mammalian cells [33], [34]. Fifty percent of Sod1 molecules represent ∼250,000 atoms of zinc. The minimum number of total zinc atoms per yeast cell required for growth is ∼5×10^6^ [17]. Thus, decreased Sod1 accumulation represents a considerable decrease (∼5%) in the total zinc demand of the cell.

Our findings may have important relevance with regard to amyotrophic lateral sclerosis (ALS), a fatal neurodegenerative disorder in humans. ALS is characterized by the progressive loss of motor neurons due to cell death. Approximately 10% of ALS cases are inherited and a subset of these are caused by dominant mutations in human Cu/Zn-SOD. While the mechanism of neuronal cell death in both heritable and spontaneous cases of ALS is not known, there is a consensus that it results from the accumulation of toxic SOD protein aggregates [35], [36]. Zinc appears to play an important role in influencing this aggregation process [37], [38]. For example, it has been shown that zinc inhibits the aggregation of both wild type and ALS mutant SOD in vitro [39], [40]. In addition, some ALS-causing mutations reduce the affinity of the SOD protein for its zinc cofactor [41]. Zinc-deficient wild type and ALS-causing SOD proteins induce apoptosis when delivered to motor neurons in vitro [42]. Finally, high dietary zinc was found to improve survival in a mouse model of heritable ALS [43]. Formation of the toxic SOD aggregates is also thought to be favored by increased oxidative stress due to the formation of intermolecular disulfide bonds [44], [45], [46]. Thus, nutritional zinc deficiency may play a role in the etiology of ALS for two reasons. First, decreased zinc availability will reduce the amount of the metal ion available for binding to the zinc site. Several studies demonstrate decreased SOD activity in zinc-deficient mammalian cells [47], [48]. Second, the increased oxidative stress associated with zinc deficiency could potentially increase the likelihood of aggregate formation.

Finally, aside from *TSA1* and *SOD1*, we did not identify any other antioxidant genes that are as important for low zinc growth. Genes playing little if any apparent role include those encoding the four other peroxiredoxins, *TSA2*, *AHP1*, *PRX1*, and *DOT5*. In addition, we found no major role for *CTT1*, which encodes the cytosolic isozyme of catalase. This result was unexpected given our recent observations indicating that *CTT1* expression is up-regulated in zinc-deficient cells and may be a direct target of Zap1 regulation [14]. No major roles were found for the glutathione peroxidases nor for transcription factors *YAP1*, *SKN7*, *MSN2*, and *MSN4* that are normally involved in oxidative stress responses. This result is consistent with our observation that the targets of these transcription factors are not induced in zinc-limited cells despite the increased oxidative stress [14], [49]. The level of oxidative stress in zinc deficiency may be insufficient to cause a response by these factors or, alternatively, their activity may be impaired under these conditions. One important caveat to these observations is that our analysis would not have detected small effects on growth rates that might have been caused by these mutations and, therefore, small but important contributions could have been missed. In addition, because we only analyzed strains carrying single mutations, we would not have identified genes that contribute to low zinc growth but are redundant with other genes. Thus, the full repertoire of genes required for tolerance of the oxidative stress of zinc deficiency remains to be defined.
